# OptiMissP: A dashboard to assess missingness in proteomic data-independent acquisition mass spectrometry

**DOI:** 10.1371/journal.pone.0249771

**Published:** 2021-04-15

**Authors:** Angelica Arioli, Arianna Dagliati, Bethany Geary, Niels Peek, Philip A. Kalra, Anthony D. Whetton, Nophar Geifman

**Affiliations:** 1 Department of Electrical, Computer and Biomedical Engineering, University of Pavia, Pavia, Italy; 2 Division of Informatics, Imaging, and Data Science, School of Health Sciences, The University of Manchester, Manchester, United Kingdom; 3 Division of Cancer Sciences, Stoller Biomarker Discovery Centre, Manchester, United Kingdom; 4 NIHR Manchester Biomedical Research Centre, Manchester Academic Health Science Centre, The University of Manchester, Manchester, United Kingdom; 5 Salford Royal NHS Foundation Trust, Salford, United Kingdom; 6 School of Medical Sciences, Faculty of Biology, Medicine and Health, Manchester Academic Health Science Centre, The University of Manchester, Manchester, United Kingdom; Aarhus University, DENMARK

## Abstract

**Background:**

Missing values are a key issue in the statistical analysis of proteomic data. Defining the strategy to address missing values is a complex task in each study, potentially affecting the quality of statistical analyses.

**Results:**

We have developed OptiMissP, a dashboard to visually and qualitatively evaluate missingness and guide decision making in the handling of missing values in proteomics studies that use data-independent acquisition mass spectrometry. It provides a set of visual tools to retrieve information about missingness through protein densities and topology-based approaches, and facilitates exploration of different imputation methods and missingness thresholds.

**Conclusions:**

OptiMissP provides support for researchers’ and clinicians’ qualitative assessment of missingness in proteomic datasets in order to define study-specific strategies for the handling of missing values. OptiMissP considers biases in protein distributions related to the choice of imputation method and helps analysts to balance the information loss caused by low missingness thresholds and the noise introduced by selecting high missingness thresholds. This is complemented by topological data analysis which provides additional insight to the structure of the data and their missingness. We use an example in Chronic Kidney Disease to illustrate the main functionalities of OptiMissP.

## Introduction

Proteomics can provide a comprehensive protein profiling of clinical and biological research samples. Recently this has enabled the discovery of proteomic biomarkers for patient stratification and disease activity. Sequential window acquisition of all the theoretical mass spectra (SWATH-MS) has enabled these advances as this data-independent acquisition (DIA) mass spectrometry technique guarantees high reproducibility in peptide identification and identifies more peptides than data-dependent methods [1]. SWATH-MS samples constituent peptides and their fragments without selection throughout the run, as opposed to being reliant largely on the selection of specific peptides for fragmentation via stochastic processes (data-dependent acquisition). It is known that less abundant peptides are harder to detect with data-dependent acquisition analysis, and therefore they are more likely to be missing [2]; there is significant literature on strategies to handle these [3–5]. On the other hand, DIA mass spectrometry lacks studies about the nature of missingness generated by this approach, as well as methods to deal with undetected peptides.

While SWATH-MS theoretically collects data on all the peptides, the resulting datasets often contain missing values. The origin of missing values in mass spectrometry is related to different events [6, 7], with both random and non-random sources. Missing values can appear at any stage from the mass spectrometric transition level up to the protein level. The specific cause for missingness varies for each individual case of missingness and depends on both biological and technical factors. In DIA mass spectrometry, the occurrence of unmeasured peptides may have either biological or technical causes; this includes sample storage, losses in protein digestion and separation, as well as failures in the measurement process. The influence of laboratory conditions, reagent lots, and personnel differences manifests itself as batch effects, which result in differences in both measured protein intensities and missing values in different batches. These phenomena give rise to data which are missing at random (MAR). In other cases, the quantification process fails because the protein’s intensity is below the detection sensitivity of the instrumentation: this generates values missing not at random (MNAR). The lack of ability to discriminate between random and non-random missingness makes the handling of missing values a difficult but nonetheless important challenge since appropriate handling is crucial to avoid bias in statistical estimates that are derived from the data. Peptides or proteins showing a high proportion of missing values can be treated in different ways: they can be excluded from further analyses, or they can be replaced by a plausible value (by imputation). Exclusion of samples or peptides with high levels of missing values from statistical analysis leads to loss of statistical power and loss of valuable data. Furthermore, it has been demonstrated that peptide values can be missing not at random in SWATH-MS data [6]. In such cases, these peptides should not be removed from the dataset because informative missingness patterns can add predictive value to proteomic signatures. Another problem when excluding proteins exceeding a certain percentage of missing values is that any missingness threshold is arbitrary. For instance, some studies use the “80% rule” [8] by which only the analytes identified in at least 80% of the samples are kept–without justification of this threshold.

Usually a thresholding strategy is complemented with the replacement of missing values using imputation methods. The choice of the most adequate imputation technique requires proper assessment of the type of missingness in the data, in order to avoid bias in the distribution of proteins and in downstream statistical analysis [9]. Various imputation approaches have been adapted to the characteristics of spectrometry data [7, 3, 10]. While there is no current consensus regarding at which level (peptide or protein) imputation is best performed, it has been shown that peptide level imputation can provide better results, with differences observable depending on the imputation methodology employed [7]. Additionally, missing values in SWATH data were also shown to be biological in nature [6]. Imputation needs to be considered on a case by case basis: there is no gold standard method to be used each time for every experiment. Each proteomics study needs to define a bespoke, detailed strategy for handling missing values, using an effective combination of thresholding and imputation that minimises the bias introduced by imputation without removing too many variables via thresholding. Such a strategy needs to be based on missingness statistics and insight into the missingness structure in the dataset. Defining the strategy is a complex and data-intensive task for which no software tools existed, until now. For this reason, we developed the OptiMissP dashboard that can assist analysts in defining study-specific strategies for handling missing values in SWATH-MS studies.

## Materials and methods

### Missingness mechanisms and imputation strategies

The OptiMissP dashboard provides users with the possibility of exploring the mechanism of missingness in a given proteomics dataset, at the protein level. Imputation methods are devoted to a specific type of missingness and so they rely on precise underlying hypotheses: missing completely at random techniques assume that the distribution of missing and complete data are the same, while MAR imputation tools are based on the idea that missing data have their own distinct distribution [11]. OptiMissP integrates five imputation methods. Four of these are commonly used approaches for handling MAR (implemented in R packages): multivariate imputations (*MICE* [12]), Random Forests (*MissForest* [13]), Probabilistic PCA, and Expectation-Maximization algorithm (*mvdalab* [14]); these have been recently evaluated as valuable imputation methods [15]. The fifth approach provides the option of exploring MNAR imputation with the lowest possible value in log-transformed data (lowest-value imputation).

### Topological data analysis (TDA Mapper)

TDA Mapper is a machine learning methodology [16] originating from the mathematical field of topology [17, 18]. Topological approaches can assist in obtaining meaningful insights into high dimensional data by extracting their shapes (networks) [16] and analysing the links between their components.

In TDA Mapper, high-dimensional data are reduced to two-dimensional data by filtering functions called *lenses* chosen to highlight specific characteristics of the data. The TDA Mapper’s output is a network with intrinsic topological features that represents the shape of data [19] in such a way that every node in a network is a cluster of instances (e.g. patients).

We propose this topological approach as a useful tool for a visual evaluation of the impact of missingness strategies on the informative content of a dataset.

While TDA is an approach robust to noise and known for preserving the intrinsic characteristics of data and the mutual relations among a subset of instances (i.e. patients), making it suitable for exploring patterns of missingness and how they correlate with other data features. Our hypothesis is that it can be a powerful method to explore how, and to what extent, different choices in term of imputations might impact on the studied datasets. Indeed, imputing data, especially in cases where missingness is informative, may modify the shape of the data, alter their intrinsic meaning and provide substantially different results.

Thus, the dashboard allows for the comparison of the topologies derived from non-imputed data with those derived from data imputed with different methods and varying missingness thresholds.

Furthermore, by enriching the TDA networks with information about the missingness in the subgroups (such as by colouring the nodes according to the mean percentage of missingness) one can determine the association of missingness and its impact on the resulting network.

TDA Mapper is sensitive to resolution parameters and the stability of its results is an object of research [20]; nevertheless it can be a resourceful tool to enhance information retrieval from data and analyse patterns of missingness.

### Proteomic data in chronic kidney disease

To demonstrate the utility of the dashboard for proteomic data, and to test its functionality, we used a SWATH-MS dataset of 410 patients with Chronic Kidney Disease (CKD) in the Salford Kidney Study, a prospective observational cohort study of patients with non-dialysis dependent Chronic Kidney Disease, who have been recruited since 2002. Any patient referred to the renal services at Salford Royal NHS Foundation Trust who is ≥18 years old with an eGFR of < 60ml/min/1.73m^2^ is eligible for recruitment. As well as routine data clinical and laboratory collection, plasma and serum samples are collected for research purposes at annual visits and stored at -80C.

All samples and data were obtained from patients with non-dialysis CKD who were managed in the secondary care renal clinical service of Salford Royal NHS Foundation trust. All patients provided written consent after face-to-face meetings with the research team. The Salford Kidney Study has been approved by the North West Regional ethics committee (REC15/NW/0818).

In this analysis the SWATH-MS analysis of the plasma samples of a proportion of the patients was performed by the Stoller Biomarker Discovery Centre as part of a project examining proteomic signals of progressive CKD. The number of detected and quantified proteins is 899. Data was acquired as previously described [21]. Briefly, non-fractionated samples were used in technical triplicate. Mass spectrometry files were acquired using a 6600 TripleTOF (Sciex, Warrington, UK). Peptide samples were introduced using an Eksigent LC system composed of a nanoLC 400 autosampler and a 425 pup module. Peptides were separated using a YMC-Triart C18 analytical column over a 120 minute gradient. The gradient was between a buffer A of 99% water, 0.1% formic acid and a buffer B of 99% acetonitrile, 0.1% formic acid. Spectra were acquired using the 100 variable winder method with locally optimized collision energy equations. SWATH data files were converted using wiffconverter (Sciex, Warrington, UK) to mzML format prior to search using openSWATH (Version 2.0.0) against a human plasma library. openSWATH result files were then scored using pyProphet before all files were aligned using the feature_alignment tool from MSproteomicstools. The requantAlignedValues tool, which performs imputation as an optional step, was not used here. Samples that had a CV of more than 20% were removed from the analysis. Features with an m_score of greater than 0.01 were also removed from the analysis. Downstream processing in R utilized the packages SWATH2stats and MSstats, using the top 3 equalise medians method, with Tukey median smoothing enabled and with imputation disabled. The output from MSstats was transformed into a data matrix of protein level information against samples, this was then used throughout.

## Results

OptiMissP is dashboard implemented via R Shiny [https://shiny.rstudio.com] with R version 3.6.1.

**Fig 1:**
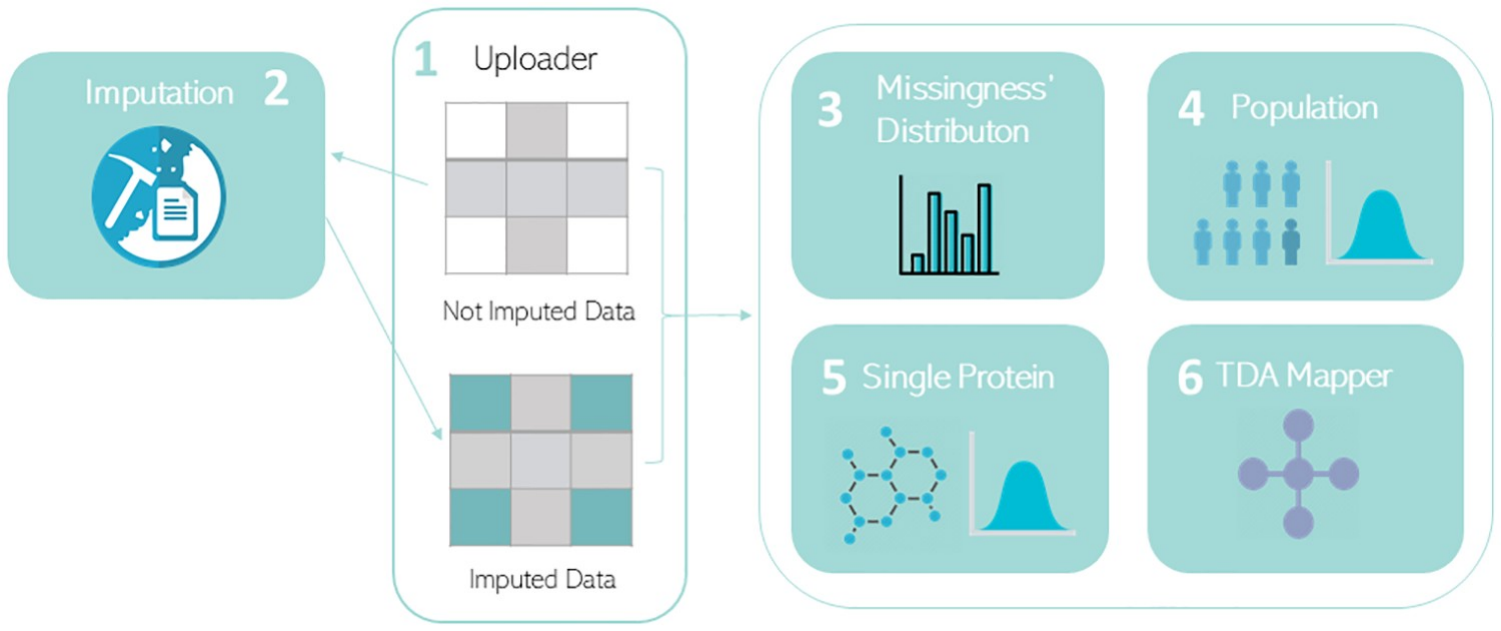
Dashboard’s architecture and components. The dashboard is composed of the six components depicted in the figure. Components 1 and 2 are dedicated to the upload the original non-imputed datasets and to the execution of the imputation step, which produces an imputed dataset that can be compared with the original non-imputed one. Components 3,4 and 5 are used for qualitative evaluation of missingness distributions and for the comparison the informative value of the imputed and non-imputed datasets. A final component (6) is used to perform TDA. Missing values imputation can be performed with any of five implemented methods: Lowest Value, MissForest, MICE, Probabilistic PCA and Expectation-Maximization algorithm. Lowest Value imputation is based on the assumption that proteins’ intensities are missing because they are too low to be detected by the instrumentation, as such, they have a biological meaning and the information of their under-expression is important. Missing values are therefore replaced by 1-value, which is the lowest possible value. MissForest, MICE, Probabilistic PCA and Expectation-Maximization algorithm, rely on the assumption that missing values are related to random events. Following different workflows, each of these methods replaces missing values with respect to the distribution in the observed data. The user can qualitatively assess the effects of imputing and thresholding (eliminating proteins with more than a threshold percentage of missingness) on the whole population and on the single protein. The evaluation tools implemented within the dashboard are distributed across the components 3,4,5 and 6. These collect visual qualitative instruments such as explorative histograms (3) of patient-level and protein-level missingness and distribution plots of imputed and non-imputed data comparatively for the whole population (4) and the single protein (5). Component 4 integrates the visual assessment tool with a quantitative analysis of the difference between the overall distribution of imputed and non-imputed data using a Kolmogorov-Smirnov test. Component 6 allows users to further refine the assessment of the imputation strategy using TDA: the user can observe how patients are distributed into the topological graphs from imputed and non-imputed data comparatively, and how missingness is related to their distributions.

As shown in Fig 1, OptiMissP is based on two components (1–2) dedicated to upload the original not-imputed datasets and to perform an imputation step, in order to retrieve an imputed dataset to compare with the original one. Three components (3–5) are used to analyse missingness distribution and compare the informative value of the imputed and non-imputed datasets and a final component (6) to perform TDA. Each of the components is described in detail as follows:

1. The Uploader serves to upload text files of non-imputed (or pre-imputed) proteomics data, at the protein level. The files may have the day in either long or wide format, i.e. instances (patients) organized into rows and features (proteins) arranged into columns or vice versa.
2. The imputation component implements the optional imputation of the data in real-time by allowing for method selection between MICE, MissForest, Probabilistic PCA, Expectation-Maximization algorithm, or Lowest Value; alternatively, the user may choose to upload a dataset already imputed.
3. Explorative histograms are aimed at assessing the global missingness in the dataset. They show the frequency of missing values from both a patient (i.e. number of missing protein values in each sample) and protein (i.e. number of samples with a missing value for each protein) perspective.
4. The fourth component presents the distribution of the mean protein intensity in the imputed versus non-imputed datasets and allows for the head-to-head visual comparison of the protein’s intensities in imputed and non-imputed data in the whole studied cohort. A slider widget allows for the selection of a missingness threshold: for the complete dataset, it filters out those with fewer missing values than the selected threshold (i.e. if the threshold is set to 80% only proteins with less than the 80% missing values in the samples are considered). As the missingness threshold is selected with the slider, density plots illustrate the two distributions (in imputed and non-imputed data) and their overlap. Distributions’ peaks (computed as maximum of value of the density function) are highlighted with a vertical dashed line. The graphic outputs are complemented with quantitative details about the number of considered proteins, the percentage of missing values in both imputed and not imputed datasets, and statistical parameters for a quantitative comparison between the distribution of imputed and not imputed data. These indicators include information about distributions’ quartiles and peaks (and their distance), and the p-value of the two-sample Kolmogorov-Smirnov test used to compare the two distributions.
5. In this section, the user can choose a specific protein of interest by its code or name (as identified in the dataset) and observe its degree of missingness and the discrepancy between its intensities’ distributions in imputed and non-imputed data. This section and results of this component are reported in S1 File.
6. The TDA Mapper component performs a two-dimensional topological analysis to show data-driven topologies built from imputed and non-imputed data. The user can decide on which TDA Mapper parameters and missingness threshold to use. Additional features enrich the resulting network with information about the missingness in each node (determined by the mean percentage) and any observed clusters in the topologies (and the options to optimize these). Identification of clusters within the TDA network is based on the *cluster_optimal* R function.

### Dashboard interface

**Fig 2:**
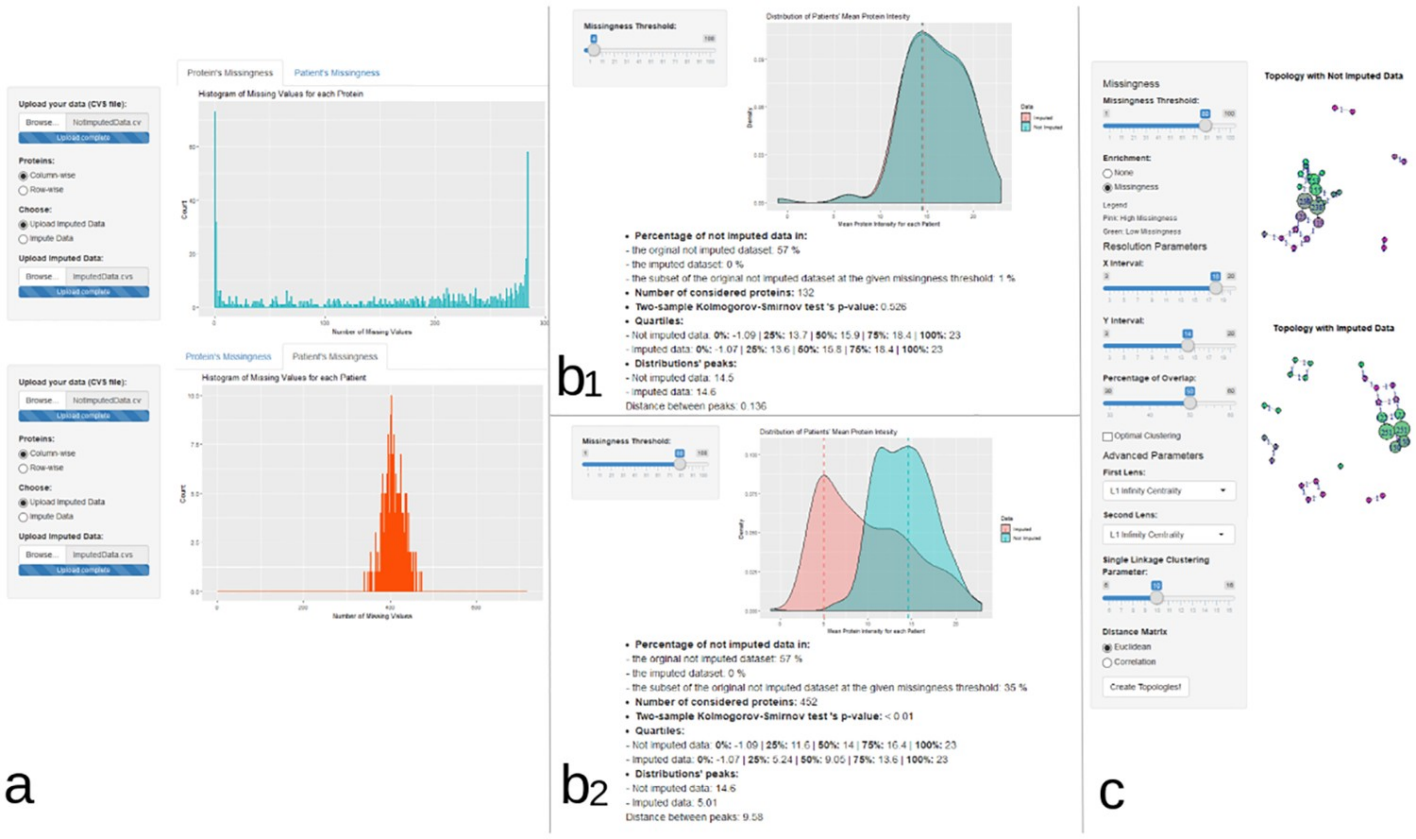
Dashboard’s principal interfaces. The principal interface of OptiMissP dashboard is shown. When applied to a dummy dataset. A) The upload of imputed and un-imputed data is shown: both datasets have been manually uploaded. Another option allows the imputation of the un-imputed dataset by choosing “Impute Data” and selecting an imputation method. Contextually, the dashboard provides two histograms showing the frequency of the number of missing values calculated for patients (instance level) and proteins (feature level). B) This section presents the comparative protein density plots for imputed and un-imputed data at two different missingness thresholds: B1, a low 4% thresholds and B2 a high 80% threshold. C) The final interface shows an example of TDA topologies for imputed and un-imputed data enriched with information about patients’ missingness.

The OptiMissP dashboard interface is illustrated in Fig 2. Data upload and imputation options are provided within the first tab (the home page of the dashboard); this section also includes explorative histograms of missingness in the data and saving options for the data that’s been imputed (Fig 2a), Once a dataset is uploaded, the subpanels positioned in the output area of the interface show the explorative histograms for proteins (top sub-panel in blue) and patients (bottom sub-panel in red). Only when both the non-imputed dataset is uploaded, and the imputation is performed–or the off-line imputed data is uploaded–do the other three functional panes appear.

The user can explore missingness thresholds with the comparative density plots of imputed and non-imputed data (Fig 2b). This allows the user to select missingness thresholds with a slider widget and shows the overlapping distributions of imputed (in red) and non-imputed (in blue) protein intensities given the selected threshold, as density plots. Within this section, it is also possible to choose a specific protein by its name (from a drop-down menu) and observe the density plots of imputed and non-imputed data for the selected protein only.

Finally, the last tab focuses on the application of TDA Mapper (Fig 2c). Here the user can re-set the thresholds of missingness, choose TDA basic (resolution and geometric scale) and advanced (function for data projection into space) parameters and run the analysis. The dashboard will display two resulting networks: the top one based on non-imputed data (i.e. the information about missingness is not included to build the shape and represent the data) and the bottom one based on imputed data. It is also possible to enrich the networks with the average missingness of each of the subjects in the TDA nodes, retrieved from the non-imputed dataset.

### A case study in chronic kidney disease

**Fig 3:**
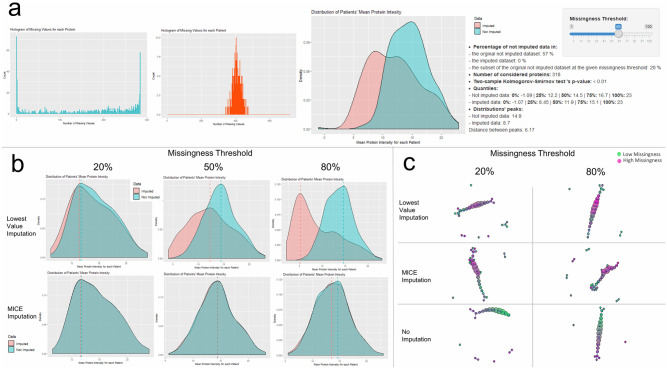
OptiMissP results in the Salford Kidney Study proteomic dataset. A) This panel presents the missingness histograms of patients’ and proteins and the density plot of patients’ mean protein intensity based on all the data with details on missingness in the text sections. B) This figure shows the density plots of patients’ mean protein intensity of imputed and not imputed data comparatively for Lowest Value (MNAR) an MICE (MAR) imputation methods and three different missingness thresholds (20%,50% and 80%). C) The section displays the results of TDA applied on not imputed data, MNAR imputed data and MAR imputed data considering two missingness thresholds. The lenses, the distance matrix and the resolution parameters are fixed. The lenses are L1 Infinity Centrality and PPCA First Component, the distance metric is the Euclidean form, the percentage of overlap is 50%, the Single Linkage Clustering’s parameter is set at 10 and the intervals are respectively 16 and 15 for the first and the second lens.

To assess the utility and usability of our tools, the Salford CKD SWATH-MS proteomic dataset’s missingness has been studied with OptiMissP dashboard. Visualization and assessment of the data shows that the 56% of the data are missing (Fig 3a) and that the missingness is normally distributed among patients and ranges from 41% to 63% of each sample’s measurements, while the number of proteins’ missing values has a peak at zero associated to the 106 proteins with no missing values (Fig 3a).

The density plots in Fig 3b are indicative of how the choice of an imputation method globally affects the informativeness of data: MAR imputation methods (in this case MICE) respect the original distribution of data, while MNAR techniques such as Lowest Value imputation introduce a visible bias, which decreases with the elimination of highly missing proteins.

As described above, it is unknown if missingness in SWATH-MS proteomic data is characterized by MNAR, MAR or whether it is a combination of both. OptiMissP allows for the handling of imputation and feature elimination with specific missingness thresholds in accordance with a qualitative analysis based on proteins value distributions.

Without this set of tools, we would have likely selected an arbitrary imputation method and missingness threshold: for example, a Lowest Value imputation and a 60%-80% threshold, as is common practice in many studies. From Fig 3b is possible to see how the OptiMissP dashboard suggests a reduction in the missingness threshold to 20%-30% when using the Lowest Value imputation approach and it confirms the choice of highest missingness thresholds (i.e. around 80% or more) for MAR methods as MICE or MissForest. Taking into consideration the information derived from this step, the TDA panel (in Fig 3c) allows for the comparison of the data shapes derived from this limited set of hypotheses and offers the possibility of studying the impact of different choices in term of imputation on a given dataset. In this example (Fig 3c), the shape of the data appears to be better conserved when the data are imputed with the Lowest Value, confirming the hypothesis of informative missingness [6].

We tested the OptiMissP dashboard functionalities in two additional publicly-available datasets with differing sizes and species [22, 23]. These results are reported and discussed in S1 File.

## Discussion

While there are numerous software tools to manage raw data from mass spectrometry (at the peptide level) [24], there is a lack of support instruments to analyse data matrices (at the protein level), especially when it comes to the problem of missingness. Few existing software packages designed for routine shotgun-style proteomics experiments utilise imputation as part of the default processing pathways. With label free quantification there are some methodologies that incorporate imputation into processing pipelines, however there is no widespread agreement on the best imputation method [10].

Data-independent acquisition proteomics has increased in use over the last decade with data imputation seeing widespread use in almost all processing software. The assumption in much of this software for data-independent acquisition is that missing values are a result of low abundance proteins and as such, methods such as imputing the lowest observed value or fixed low numbers are employed. MSstats, for example, has been utilised for both imputation and protein summarisation in both label free DDA approaches and data-independent acquisition, and by default uses an accelerated failure model after median normalisation [25].

We present here a dashboard for the visualization and exploration of missing values in high throughput pre-processed proteomics data. The dashboard includes a set of tools aimed at guiding researchers in the handling of missingness within their studies, allowing for the identification of an initial strategy based on a qualitative evaluation of missingness by comparing different imputation approaches.

Using data from the Salford Kidney Study CKD proteomic dataset, we demonstrate the utility of the OptiMissP dashboard. In this example, exploration of missingness in the data suggests that a MAR imputation method is preferable, as is applying a high missingness threshold (i.e. 90% threshold). Given that the enrichment with the patients’ percentage of missing values indicate different patient distributions within the different TDA networks (Fig 3c), and the substantial overlapping of the distributions in data imputed with the Lowest Value and with MICE when a threshold of 20% is used (Fig 3b), OptiMissP suggests a possible concurrent effect of MAR and MNAR. In order to better assess these effects, and eventually separate them, further evaluations are required, which must also include information about repeated experiments or experiments performed in different laboratory settings.

The OptiMissP dashboard is aimed at supporting a visual and intuitive assessment of missingness in a user-friendly manner; it is explainable and easily interpretable by users, as opposed to providing optimized solutions achieved by black-box algorithmic calculations.

As recently discussed [26], different studies (e.g. risk prediction studies or parameter estimation researches) may require different combinations of imputation method and missingness thresholds, which depend on various factors including the scope of the prediction model (if causally principled or aimed at maximizing information), the model development stage, and casual considerations. In the context of DIA, our qualitative tool support can be used to tailor the initial phases of a study to a specific purpose and balance the bias introduced with missing values’ handling strategies.

We acknowledge that root-mean-square error (RMSE) analysis would be the most appropriate approach to compare how imputation approaches handle missingness. However, since it is likely that complete observations (i.e. samples with no missing values) are not always available, as is the case in many SWATH proteomics datasets, that strategy was not feasible. Instead, we relied on the distance between peaks of the distributions, and use Kolmogorov-Smirnov test to compare imputed and non-imputed data sets. Future developments should include the RMSE computation. Additional developments of OptiMissP will be devoted to evaluating the usability and potential uptake of the tool by naive end-users. We will further enhance the tool with functionalities aimed at defining thresholds and imputation methods on a quantitative basis for multivariate prediction model validation and deployment [27]; as well as explore methods for handling missingness at the transition/peptide level. Further efforts will be also dedicated to include the option of performing enrichment-based analysis based on proteins’-related pathways.

## Conclusions

OptiMissP can aid research in addressing the issue of missingness in mass-spectrometry proteomics studies, supporting statistical analyses at the basis of proteomics biomarker discovery. We have implemented a data-driven visual process to explore missingness aimed at finding a balance between the removal of a great amount of informative data (i.e. choosing high thresholds of missingness) and the introduction of biases in protein distributions (i.e. using imputation methods that do not reflect the nature of the missingness). The dashboard fits into this context by enhancing clinician and researcher abilities to make better, informed decisions with respect to handling missing values, and retrieving information from missingness itself.

## Supporting information

S1 File (DOCX)

## Abbreviations

CKD: Chronic Kidney Disease
DDA: data-dependent acquisition
DIA: data-independent acquisition
eGRF: estimated glomerular filtration rate
MAR: missing at random
MCAR: missing completely at random
MNAR: missing not at random
ND-CKD: non-dialysis dependent Chronic Kidney Disease
SKS: Salford Kidney Study
SWATH-MS: sequential window acquisition of all the theoretical mass spectra
TDA: Topological Data Analysis
