# Supplementary material for: OptiMissP: A dashboard to assess missingness in proteomic data-independent acquisition mass spectrometry
Source: PLoS One. 2021 Apr 15;16(4):e0249771. doi: 10.1371/journal.pone.0249771 (PMC8049317; doi:10.1371/journal.pone.0249771)
Supplement: S1 File — (DOCX) [file pone.0249771.s001.docx]

**S1 File**

***A. Single protein section***

The following screenshot reports the section of the dashboard dedicated to the analysis of single proteins. In this case proteins were identified by their UniProt ID. Images reports proteins found to be relevant to distinguish CKD patients’ classes: Transgelin-2, IgGFc-binding protein, and Laminin subunit alpha-5


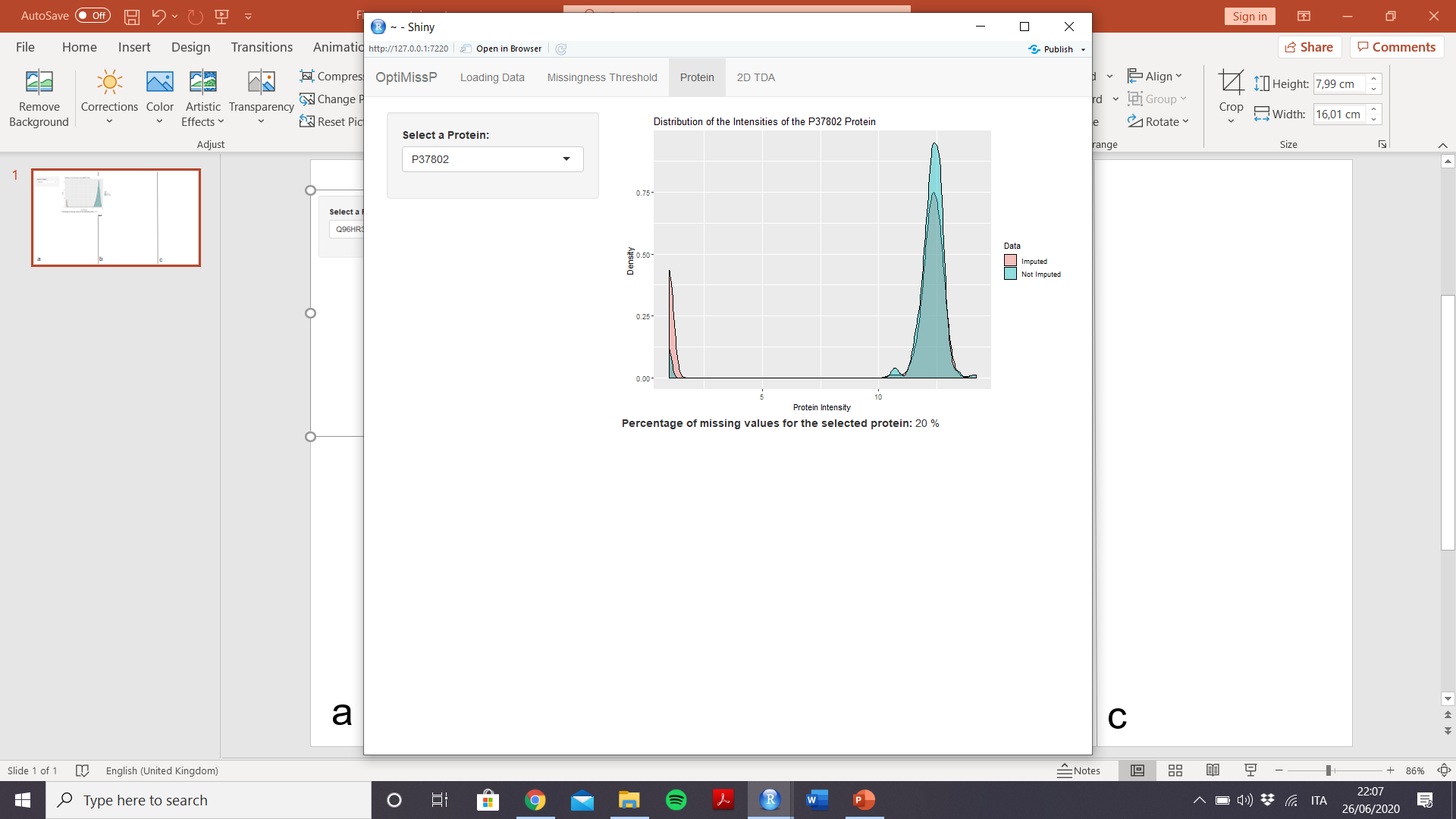


**S1 Fig**. Transgelin-2 protein plot


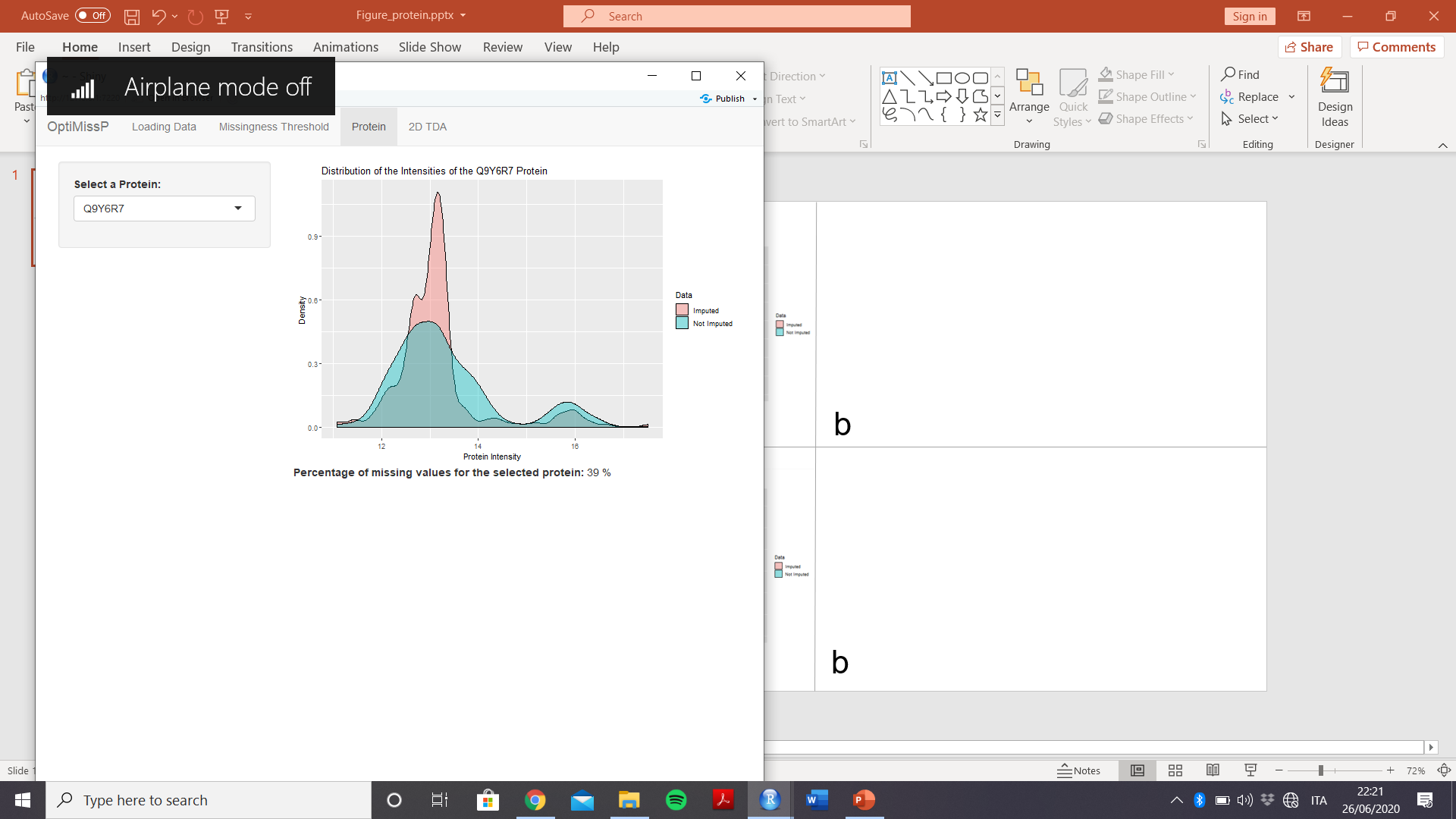


**S2 Fig**. IgGFc-binding protein plot


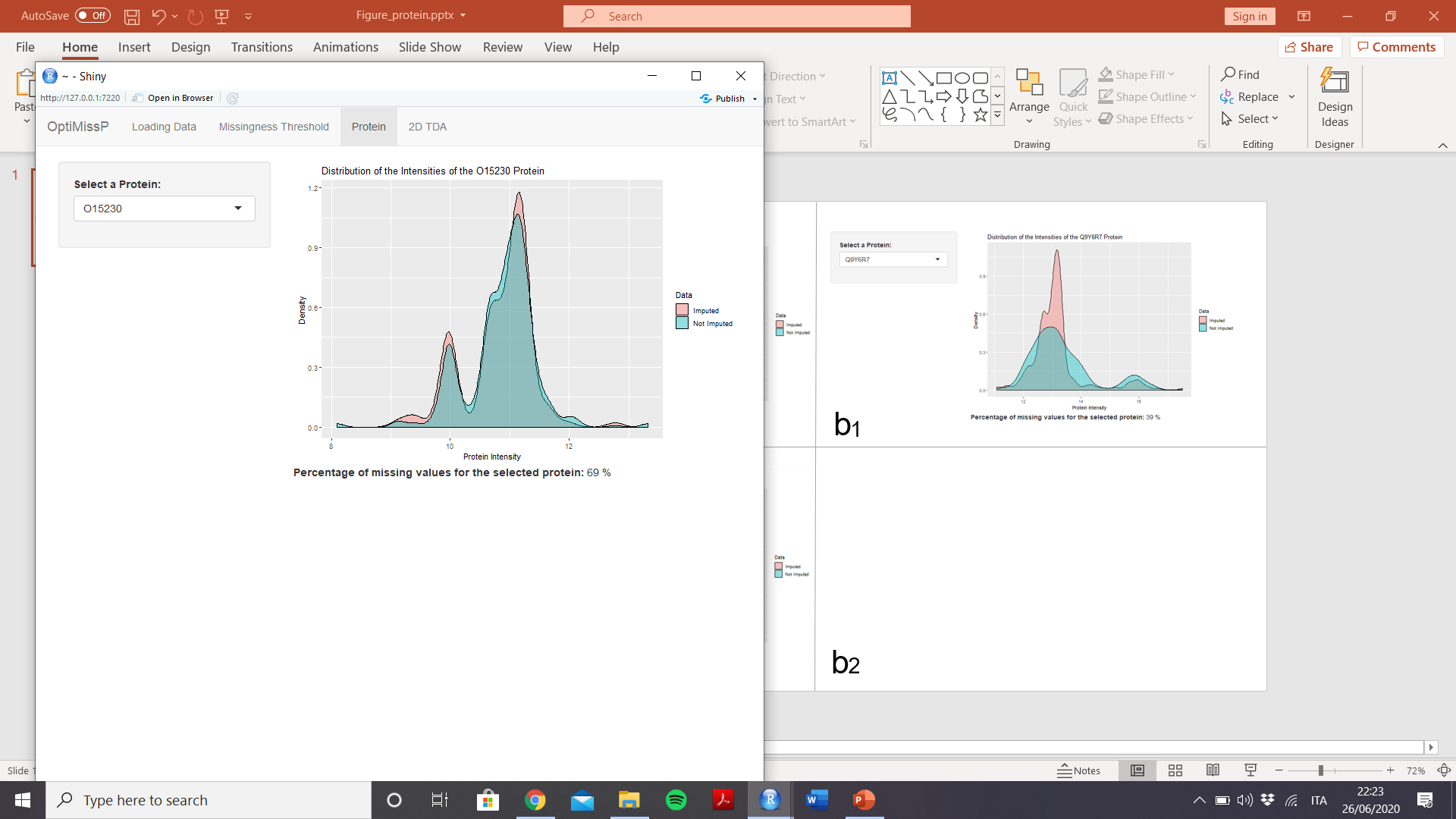


**S3 Fig.** Laminin subunit alpha-5 protein plot.

***B. Additional Datasets***

In the following we report results obtained when applying OptiMissP to two additional open, publically available dataset that differ from the one presented in the main manuscript in terms of size (they include substantially smaller populations), pathology of interest (liver cancer, and HIV) and species (human and mouse).

The first analyzed dataset include data from proteome analysis of human immunodeficiency virus (HIV)-1-infected (8 samples) and it is available in the Pride archive with ID PXD005234 (<https://www.ebi.ac.uk/pride/archive/projects/PXD005234>). In this study proteome maps were generated by SWATH-MS and indicate a range of functionally changes in the proteome of HIV infected human CD4+ T cells. More specifically, the experiment consisted in a SWATH-MS approach to measure the proteome of human primary CD4+ T cells infected with HIV-1 in vitro as well as CD4+ T cells from HIV-1 infected patients with paired samples on and off antiretroviral treatment. Within the study it was possible to consistently detect 895 proteins.


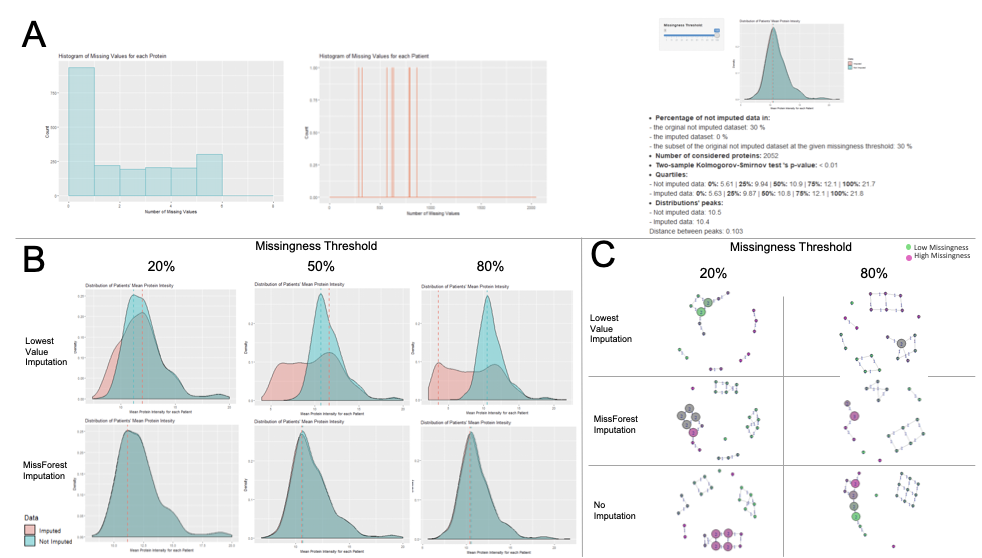


**S4 Fig.** OptiMissP results in analyzing data from proteome analysis of human immunodeficiency virus. A. missing data distributions, B. missingness thresholds, C. TDA representation.

The second analyzed dataset include data from application to a mouse liver cancer model (20 samples) and it is available in the Pride archive with ID PXD008758 (<https://www.ebi.ac.uk/pride/archive/projects/PXD008758>). In this study authors applied an original strategy to a panel of wild-type and mutant mice that are either healthy or present liver cancer in order to detect proteome changes based on mtES scores and Overall to provide a sensitive approach to cancer biomarker discovery that takes into account contextual information of tested proteins. Within the experiment, authors identified and quantified over 2700 proteins by using SWATH-MS.


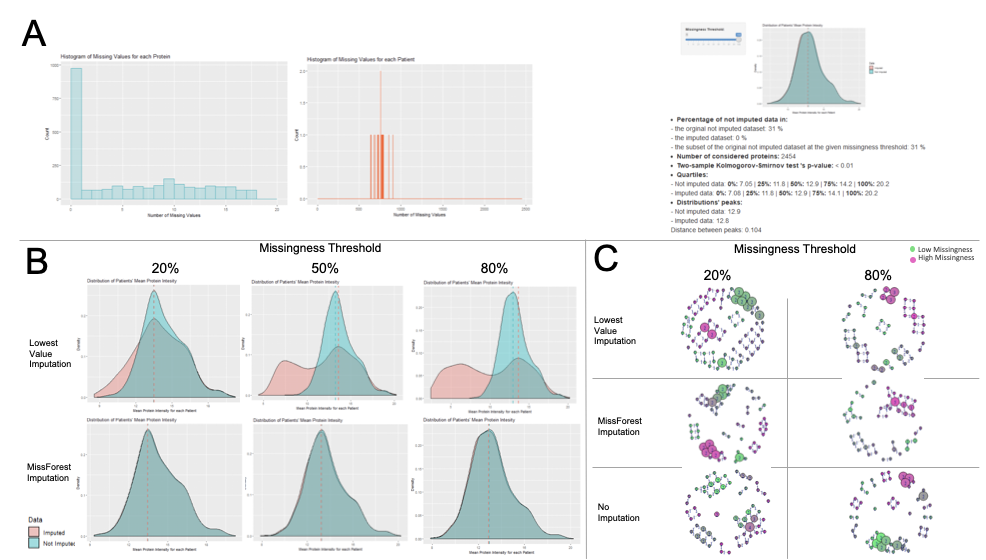


**S5 Fig.** OptiMissP results analyzing data from application to a mouse liver cancer model. A. missing data distributions, B. missingness thresholds, C. TDA representation.

In both cases it is possible to observe the suitability of missingness thresholds of 20% when using the Lowest Value imputation and highest missingness thresholds (i.e. around 80% or more) for MAR methods as MICE or MissForest, confirming previous results and considerations on the nature of missingness in DIA.

As expected, TDA results were sensitive to the different information contained in the data, but also sensitive to the sample size reduction. However - especially in the second dataset – it can clearly be seen how the topology is able to well separate observation on the basis of high/low levels of missingness.
